# Primary Care EHR data on Social Determinants of Health: Quality and Fitness for Purpose in Precision/Personalised Medicine

**DOI:** 10.1055/s-0044-1800716

**Published:** 2025-04-08

**Authors:** Anthony Paulo Sunjaya, Myron Anthony Godinho, Jitendra Jonnagaddala, Craig Kuziemsky, Karen Tu, Rafiqul Islam, Tasuku Okui, Naoki Nakashima, Javier Silva-Valencia, Leonardo Rojas-Mezarina, Alvin Marcelo, Sabrina Wong Kay Wye, Chien-Yeh Hsu, Uy Hoang, Jack Westfall, Simon de Lusignan, Siaw-Teng Liaw

**Affiliations:** 1 The George Institute for Global Health, UNSW Sydney, Australia; 2 Westmead Applied Research Centre, Faculty of Medicine and Health, University of Sydney, Australia; 3 School of Population Health, UNSW Sydney, Australia; 4 MacEwan University, Edmonton, Alberta, Canada; 5 University of Toronto, Ontario, Canada; 6 Kyushu University, Japan; 7 Telehealth Unit Department, National University of San Marcos, Lima, Peru; 8 University of the Philippines, Manila, Philippines; 9 Clinical Research Unit, Nanyang Technological University, Singapore; 10 Taipei Medical University, Taiwan; 11 Department of Primary Care Health Sciences, University of Oxford, UK; 12 University of Colorado, USA

**Keywords:** Primary Care, Electronic Health Records, Social Determinants of Health, Precision Medicine

## Abstract

**Introduction** : Precision and personalised medicine requires comprehensive genetic, epigenetic, lifestyle, social, community and environmental knowledge of the patient. This approach highlights the importance of the social determinants of health (SDoH), described by the World Health Organization (WHO) as ‘the non-medical factors that influence health outcomes, the conditions in which people are born, grow, work, live, and age, and the wider set of forces and systems shaping the conditions of daily life such as economic policies and systems, development agendas, social norms, social policies and political systems’.

**Methods** : This study examined if countries collect SDoH indicators and, if they do, the quality of the data and whether they are fit for clinical and population health purposes. The sources of data were EHR networks and, where not available, national data collections.

**Results** : While demographic details (age, gender) and rurality were well documented in most countries, we found that data availability and quality for education, occupation, income, socio-economic status, and residential care varied considerably between countries. Data for smoking, obesity, alcohol use, mental health, and substance use were generally poorly recorded.

**Conclusion** : Recommendations include a universal set of indicators and taxonomy for SDoH; common data model and metadata standards for national and global harmonisation and monitoring; benchmarks for data quality and fitness-for-purpose; capacity building at national and subnational levels in data collection, data analysis, communication and dissemination of results; ethical and transparent data stewardship; and governance, leadership and diplomacy across multiple sectors to co-create an enabling policy and regulatory environment.

## 1. Introduction

Precision and personalised medicine requires comprehensive genetic, epigenetic, lifestyle, social, community and environmental knowledge of the patient. This approach is consistent with the biopsychosocial model of primary care, general practice and family medicine, and highlights the importance of the social determinants of health (SDoH). At the level of communities and neighbourhoods, health outcomes have been linked to several SDoH-related factors, including: crime and safety; education; employment and income; health and social services; housing; leisure and culture; local food and other goods; natural environment; public open space; transport; and social cohesion and local democracy [ 1 ]. In other studies, a broad range of social factors were found to be associated with the risk of post-discharge readmission and mortality in patients with pneumonia and heart failure (HF), including low socio-economic status, living situation, lack of social support, being unmarried and risk behaviors. Similar findings were observed for factors associated with mortality after HF, along with psychiatric comorbidities, lack of home resources and greater distance to hospital [ 2 ]. The SDoH account for 80–90% of health outcomes and encompass factors such as: health related behaviours (such as physical activity, diet, smoking, alcohol consumption), socio-economic factors (such as employment, access to adequate housing, access to transport) and environmental factors (such as access to clean air, clean water) [ 3 , 4 ].

In establishing the Commission on Social Determinants of Health in 2005, the World Health Organization (WHO) described social determinants as ‘the non-medical factors that influence health outcomes [ 5 ]. They are the conditions in which people are born, grow, work, live, and age, and the wider set of forces and systems shaping the conditions of daily life. These forces and systems include economic policies and systems, development agendas, social norms, social policies and political systems’ [ 6 , 7 ]. While many countries have committed to addressing SDoH and improving health equity, policy and practice has been slow and uneven, with limited focus on key structural determinants, such as inequitable economic systems, structural discrimination including intersecting racism and gender inequality, and weak societal infrastructure.

A key challenge facing governments is the development and implementation of effective policies and interventions and the assessment of their impact on health equity. This requires monitoring of SDoH and their association with health inequities, as well as monitoring of policies and interventions addressing SDoH and health equity. However, many countries lack the data, information systems and capacity to monitor progress on SDoH and related policies.

### 1.1. EHR data quality and the Social Determinants of Health

To enable general practice and family medicine to incorporate the SDoH in clinical practice, the supporting primary care information system and ecosystem must be based on this multidisciplinary and multisectoral model of care and must collect data appropriate to meet this information need. Electronic health records (EHRs) are the primary vehicle by which data on the SDoH are collected, stored, and acted upon. EHRs provide crucial information to:

- providers treating individual patients;
- health systems, including public health officials, about the health of populations;
- researchers about the determinants of health and the effectiveness of treatment.

Inclusion of social and behavioral health domains in EHRs is vital to all three uses [ 8 ]. However, primary care EHRs do not routinely include SDoH in the EHR architecture. Nonetheless, the following are usually included in EHRs and may be useful indicators or proxy measures for SDoH:

- Age;
- Gender;
- Ethnicity, e.g., using WBAMO (White, Black, Asian, Mixed, Other) with subcategories;
- Socio-economic status, measured using IMD, e.g., income, education;
- Rurality, measured using population density and rural, Town & City conurbation;
- Regionality, differences among regions and between global north and global south;
- Obesity, based on BMI in adults;
- Smoking status, sometimes alcohol, residential care & others.

However, EHRs are widely believed to lack fit-for-purpose data on the SDoH [ 9 ]. This paper will therefore focus on the informatics support currently provided by a selection of global health information systems for enabling the social dimensions of the biopsychosocial model.

### 1.2. Objectives

- To report on national taxonomies for SDoH that could be applied in the design of EHRs in participating countries;
- To report on the quality of SDoH indicators collected in EHRs, and comment on their fitness for purpose in participating countries;
- To make recommendations to improve the quality of SDoH indicators, including those collected in other public health systems in participating countries.

## 2. Methods

Representatives from member countries of the International Medical Association's (IMIA) Primary Care Informatics Working Group – Australia, Canada, Japan, Peru, Philippines, Singapore, Taiwan, United Kingdom (UK) and United States of America (USA) - participate in the study. A standardised data collection template was used collect, within existing ethical approval constraints, the following data:

Contextual information on policy and SDoH taxonomy of key indicators – SES, food, housing and environment, early childhood, discrimination, social, access and equity, and social prescribing ( Table 1 );

**Table 1.** Social Determinants of Health Taxonomies used in participating countries.

SDoH taxonomy	Participating country								
	Australia	Canada	Japan	Singapore	Peru	Philippines	Taiwan	UK (England)	USA
SES Socioe-conomic status (SES) is often described through indicators such as income, educational attainment, or level of occupation	Income not captured in EHRs. Social protection factors include social inclusion (not captured in EHRs) and family relationships. The federal government (AIHW) has oversight of SEIFA, the Socioeconomic Index for Area, is calculated and related to postcodes.	Income quintile derived from postal codes using Stats Can postal code conversion file https://www150.statcan.gc.ca/n1/en/catalogue/82F0086X Marginalization indices with 4 dimensions (residential instability, economic dependency, ethno-cultural composition & situational vulnerability)) are derived from postal codes 1	Captured in EHR as income group (Only for elderly patients or patients who used High-Cost Medical Expense Benefit).	Actual income level is not captured. Instead, housing type captured in the EHR provides an estimate of socioeconomic status.	Captured as “Economic Situation” which takes possible values using the minimum wage as a reference (e.g. <minimum wage, minimum wage, 2x minimum wage, 3x minimum wage)	“Monthly income”	Captured in EHR as income group. SES data are hosted and integrated in Center for Health & Welfare Information Science, Ministry of Health and Welfare, Taiwan.	Captured in EHR as part of the Index of Multiple Deprivation (IMD) Score based on patient location (postcode). https://www.gov.uk/government/statistics/english-indices-of-deprivation-2019	% of poverty level US adjusted by area (postcode). Standardized approaches not collected in the two most common SDoH instruments, instead the foci are on food and housing security – which are typically tied to income
	Education not captured in EHR but may be in text or semi-structured fields.	Education not formally captured in EHR but may be in text or semi-structured fields.	Education not captured in EHR but may be in text or semi-structured fields.	Education not captured in EHR but may be in text or semi-structured fields.	Captured as a categorical variable called grade of instruction	“Highest educational attainment”	Not captured in EHR. Data available from Statistical Dept of Ministry of Interior	IMD score	Included in some surveys but not all. How education is recorded varies by instrument.
	Occupation captured, but job insecurity is not.	Occupation or employment status may be captured in semi-structured fields	Not captured in EHR	Captured in EHR as “Employment”	Captured as a text field.	“Occupation description”	Captured in EHR as “Employment”	IMD score	Included in some but not all instruments directly
	Working life conditions not captured as part of EHR in structured codable fields.	Working life conditions not captured in any formal way	Working life conditions not captured as part of EHR in structured fields. M Statistics Canada. (2023). The Canadian Index of Multiple Deprivation, 2021. Statistics Canada Catalogue no. 45-20-0001. ay be inferred from occupation?	Working life conditions not captured as part of EHR in structured codable fields. May be inferred from occupation?	Captured in a separate form called Family File. Possible values: Stable Worker, Temporary, without occupation, Retired, Student	“Point of service financially incapable” and “Financially incapable”	Working life conditions not captured as part of EHR in structured codable fields. May be inferred from occupation?	Not captured in EHR	Not typically included
SES or Deprivation Indices	Inferred from SEIFA	Income quintile derived from postal codes using Stats Can postal code conversion file https://www150.statcan.gc.ca/n1/en/catalogue/82F0086X Material and social deprivation (Canadian Index of Multiple Deprivation)	Not captured in EHR	Marital status	Not captured	Not captured		IMD score	
Food	Food insecurity not captured in EHR	Social inequities	Not captured in EHR	Acknowledged as important, but no national definition or data collected and monitored currently.	Food insecurity not captured	Food security captured in Pantawid Pamilya Information System	Food insecurity not captured in EHR	Food insecurity not captured in EHR	Included in all common instruments
Housing & environment	Housing captured as type of living arrangements. However, basic amenities & home environment not captured in EHR	Household ownership captured. Physical and Social Environment may be captured & monitored as “Sense of community belonging” (somewhat strong or very strong) or “Living alone” Residential instability captured as: Proportion of dwellings that are apartment buildings, Proportion of persons living alone, Proportion of dwellings that are owned, proportion of movers within the past 5 years, Proportion of the population that is married/common-law, Median 2021 household income	Household ownership captured	Captured in EHR as “Housing type”	Captured in a separate form called Family File. Questions include: basic amenities, material of the house, available ways to conserve food, own transportation, dispose of garbage, environment risk (floods, landslides, etc), animal ownership.	“Household unit”	Household ownership captured	Captured in EHR as part of the Index of Multiple Deprivation (IMD) Score based on patient location.	Included in all common public health instruments
Early childhood The domains of early childhood development – (physical, social, emotional, language & cognition) strongly influence school success, economic participation, social citizenship and health.	Early childhood development typically captured via the “Blue Book” template which include the Denver Development Scale in the EHR. Participation in specific projects e.g. Best Start, First 2000 days may involve a specific EHR template	Typically captured in a standardized format with regular ‘well baby visits’ in Rourke records ( https://www.rourkebabyrecord.ca/rbr2020/default ). This includes a Nipissing test at 18 months ( https://www.peelregion.ca/health/professionals/pdfs/ndds-18-months-2011.pdf ).	Not captured in EHR	Early childhood development monitored by the Ministry of Social & Family Development (low income families, or children with speech and language, social skills, motor skills or behavioural needs)	Captured in a separate form called Family File. Includes questions as: premature birth, visual and hearing assessment competition, complete vaccines, supplement regimen (Iron, micronutrients).	“Integrated Maternal and Child Illness”	Early childhood development	Not captured in EHR	Part of routine childcare but not considered part of SDoH activities. Several different screening approaches are used
Discrimination	Structural conflict 2 being addressed by legislation and education & training in social inclusion & non-discrimination	Physical and Social Environment (Sense of community belonging, somewhat strong or very strong is one indicator tracked)	Not captured in EHR	Workplace discrimination monitored by Ministry of Manpower	Captured as a separate questionnaire about history or signs of abuse or negligence. Different questionnaire for infants.	Person with Disability Identifier	Physical and Social Discrimination	Not captured in EHR	Not a part of routine instruments in the US
Social	Not captured in EHR but may be inferred from living arrangements	“Lives alone” is one indicator tracked	Not captured in EHR but may be inferred from living arrangements	“Lives alone” is captured in the EHR as well as living arrangements	Not captured	Not captured	Captured as “Social benefit”	Not captured in EHR	Intimate Partner Violence (IPV) is included in all common instruments but not general social conflict
Access & equity	Australia has a universal healthcare insurance system with GP services funded federally & hospital-based & community health services funded through the states.	Canada has a universal healthcare system that provides access to all via provincially organized and administered services e.g. physician visits, emergency room visits, hospitalizations, drug coverage (extent population covered varies by province)	Japan does not have a universal and free public health system but has specific programs for the elderly and low SES groups	Government -funded primary care services are available universally with low out of pocket payments, and additional coverage for elderly, children and low SES groups. There is open access to primary care services i.e. can walk into a primary care clinic and be seen on the same day	Nearly all Peruvian citizens have health insurance - Public sector covers >70% of citizens; EsSalud subsystem covers formal workers & their families (30% citizens), Police & armed forces subsystems, and private sector.	Patient assignment to a healthcare facility	Taiwan's national health insurance is provided to 99.9% of citizens and residents	Not captured in EHR	Transportation and some access issues are included but not this construct specifically
Social prescribing	The green or lifestyle prescription is not captured in the EHR but may be in the text or semi-structured fields.	The green or lifestyle prescription is not captured in the EHR but may be in the text or semi-structured fields.	Not captured in EHR	Lifestyle prescription monitored by Ministry of Health (Healthier SG)	Not captured	Not captured	Social Prescribing	Captured in social prescribing dashboard for RCGP RSC sentinel surveillance practices. https://www.rcgp.org.uk/representing-you/research-at-rcgp/research-surveillance-centre#practice	Not part of SDoH issues in the US
Source / Data custodian	AIHW SDOH https://www.aihw.gov.au/reports/australias-health/social-determinants-of-health The SW Sydney electronic Practice Based Research Network (ePBRN) 2019 Linked Dataset v2e is governed by the SREDH ePBRN WG ( https://www.sredhconsortium.org/sredh-datasets/epbrn-2019-gp-hospital-linked-dataset ) It includes linked data from 3 EHR systems, 11 GP sites & 5 hospitals). 158,159 subjects from 8,909 sites.	Income quintile derived from postal codes using Stats Can postal code conversion file https://www150.statcan.gc.ca/n1/en/catalogue/82F0086X https://www150.statcan.gc.ca/n1/pub/45-20-0001/452000012023002-eng.htm is the website or you can use this reference Statistics Canada. (2023). The Canadian Index of Multiple Deprivation, 2021. Statistics Canada Catalogue no. 45-20-0001 https://health-infobase.canada.ca/health-inequalities/Index is the website or you can use Pan-Canadian Health Inequalities Data Tool. A joint initiative of the Public Health Agency of Canada, the Pan-Canadian Public Health Network, Statistics Canada and the Canadian Institute for Health Information. Available from: https://health-infobase.canada.ca/health-inequalities/Indicat . Canada has a health inequalities data tool with >160 health determinant & outcome indicators groups into 14 domains.	Statistical Bureau of Japan Ministry of Internal Affair and Communications https://www.stat.go.jp/english/	Department of Statistics Singapore https://www.singstat.gov.sg/publications/reference/ebook Ministry of Social and Family Development https://www.msf.gov.sg/research-data/research-reports-data Ministry of Manpower https://stats.mom.gov.sg/iMAS_PdfLibrary/mrsd-Fair-Employment-Practices-2022.pdf Ministry of Health (Healthier SG) https://www.healthiersg.gov.sg/resources/white-paper/	Data hosted in each health institution or health network. The RENHICE Law for the interoperability of EMRs supports a centralized EMR model managed by Ministry of Health. But interoperability platform has not been fully implemented and >70% of healthcare facilities still maintain a mix of paper & EMRs. The MOH General Office of Information and Technology manages & integrates summary data from the public sector, and published as statistical information for general use.	DOH Joint Administrative Order 2021-0002 (bit.ly/mandatoryadoption) DSWD Pantawid Pamilya Pilipino Program or 4Ps Operations Manual)	Center for Health & Welfare Information Science, Ministry of Health and Welfare, Taiwan: https://dep.mohw.gov.tw/DOS/lp-2506-113.htmlhttps://dep.mohw.gov.tw/DOS/lp-2506-113.html Statistical Dept of Ministry of Interior: https://www.moi.gov.tw/cp.aspx?n=5590 and https://www.moi.gov.tw/cl.aspx?n=646	RCGP RSC sentinel surveillance network/ University of Oxford. https://www.rcgp.org.uk/representing-you/research-at-rcgp/research-surveillance-centre EHR from over 1900 general practices (17,299,780 patients) in the English National Sentinel Network. https://www.ncbi.nlm.nih.gov/pmc/articles/PMC9770023/ https://www.ncbi.nlm.nih.gov/pmc/articles/PMC9359118/	Selected payers and granting agencies require reporting but it is not universal. eCW and EHR data from medical facilities- all different specialties- (care sites) all around the USA (50 states, DC & 2 territories
SDoH taxonomy Meta-data									
FAIR Guiding Principles Findable, Accessible, Interoperable & Reusable	There is a formal process to access and use large data sets under government (AIHW) custodianship. The MeTEoR provides come consistency of datasets but generally FAIR is not fully implemented in Australia. Data linkage is being done within the auspices of the AIHW. The AIHW is also the custodian of the data from My Health Record.				Peru's Ministry of Health uses an Open Data platform, which curates and publishes disaggregated anonymous clinical data. However, the publication is not systematic, and publishes domain-specific databases e.g. COVID-19, vaccination, anaemia. https://www.datosabiertos.gob.pe/ Findable: The data is assigned a unique identifier. Information published in Open Data contains metadata including identifier, publisher, date and license. Accessible: Clinical Data is shared partially, anonymized, and only related to certain topics: COVID-19, vaccination, anemia, etc. All as CSV files in the Open Data platform: https://www.datosabiertos.gob.pe/ Interoperable: No interoperability between other institutions. Reusable: Information published in Open Data contains metadata including identifier, publisher, date and license.	DSWD has a Data Privacy Policy (based on the Data Privacy Act of 2012) -- but no FAIR principles.			Not even considered in EHRs. Health insurance datasets use ICD-9 or ICD-10?
Risk management framework	Five Safes: Safe people, project, data, setting, & outputs				There are security standards and regulations that health facilities must follow when implementing electronic healthcare records, especially certain aspects related to health data protection. (Law 29733: Data Protection Law, RM 688-2020: Personal Health Data)	Risk levels Zero, low, medium, high (Pantawid Pamilya Operations Manual)		Safe people, project, data, setting, & outputs through the trusted research environment. https://www.hdruk.ac.uk/access-to-health-data/trusted-research-environments/	Covered under general privacy rules nothing specific to SDoH
Ethics approval required?	Yes, the data users already have pre-approved ethics to share the submitted data. The ethics was approved by UNSW Sydney & SW Sydney Local Health District Human Research Ethics Committees (Project #: 2019/PID05368)					N/A		Yes	
Data sharing & governance arrangement?	Governance for ePBRN is managed through the SREDH Consortium ePBRN working group ( https://www.sredhconsortium.org/sredh-working-groups/electronic-practice-based-research-network ).	Governance for UTOPIAN/INTREPID https://www.intrepidprimarycare.org/resources				None		Governance for this dataset is managed through the RPIMDISC approval committee https://orchid.phc.ox.ac.uk/using-orchid-for-research	https://www.aafp.org/family-physician/patient-care/nrn/studies/all/dartnet.html

1 Statistics Canada. (2023). The Canadian Index of Multiple Deprivation, 2021. Statistics Canada Catalogue no. 45-20-0001.

2 Structural conflicts are deep-rooted societal issues ingrained within the structure or framework of society and include (but aren't limited to) racism, sexism, classism, ableism, xenophobia, and homophobia. These conflicts restrict access to education, employment, and healthcare services, worsening health disparities.

Summary data from existing digital data repositories, both derived and aggregated from EHRs or specific-purpose national data collections ( Table 2 );

**Table 2.**
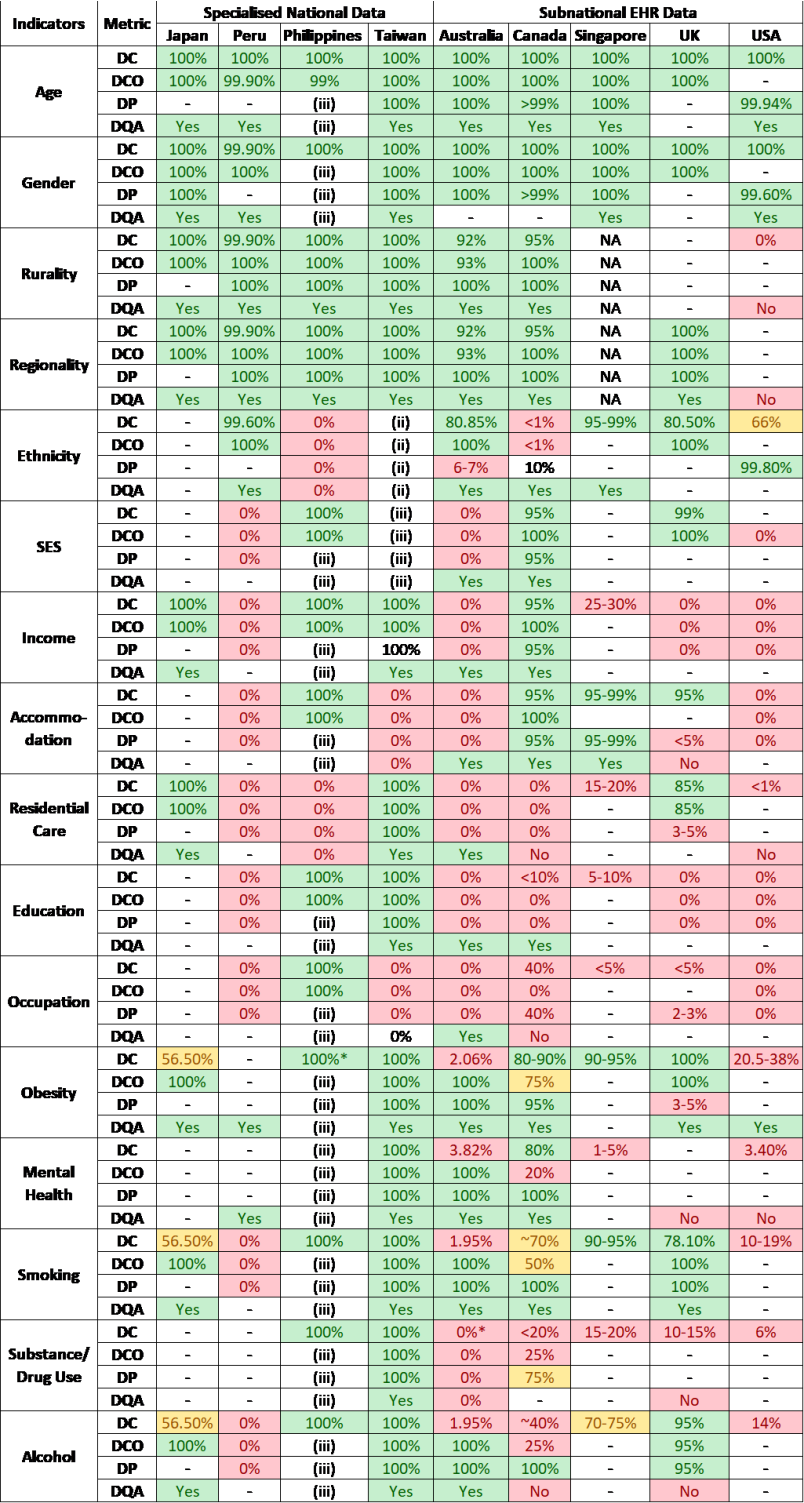
Data completeness, conformance to a taxonomy, plausibility, and availability of prior quality assessment from the participating countries. DC - Data Completeness (%), DCO – Data Conformance (as a percentage of available data only), DP – Data Plausibility(%), DQA – Formal Data Quality Assessment Availability (Yes/No). Legend (‘traffic light’ colour coding):

Report the quality and fitness for purpose of the data collected, using the completeness, conformance and plausibility framework [ 10 , 11 ], and whether a formal data quality assessment is routinely done or have been reported ( Table 2 ).

Data for the following SDoH indicators were prioritised for collection - Age, Gender, Ethnicity, SES, Income, Accommodation, Education, Occupation, Obesity, Mental health, Smoking, Alcohol, Residential care, Rurality, Regionality, and LMIC status. There was also the possibility of submitting data for other indicators present in the databases where the data was sourced from.

The Kahn *et al.* framework [ 10 ] with an enhancement to cover extrinsic factors across the data lifecycle [ 11 ] was used to assess data quality and fitness for purpose. The categories of data quality are completeness, conformance to standards and benchmarks, and plausibility, which may be temporal or atemporal. This framework also guided the development of a data quality dashboard for the global OHDSI (Observational Health Data Science Informatics) community [ 12 , 13 ]. Completeness was assessed against all the data potentially available in the EHR dataset. The conformance and plausibility were assessed against the data available as the denominator; this was adopted because of uncertainty about the data collection, extraction, and documentation methods in the data collections examined. We classified the data quality into three categories : Red, if quality <50%; Orange, if between 50-75%; and Green if >75%. This “traffic light” categorisation is presented in Table 2 . Data details are available as Supplementary File.

## 3. Results

### 3.1. Overview

Data from nine countries – five from the Asia-Pacific region, two from North America, and one each from Europe and South America were analysed. These countries represented a variety of health system models including those where healthcare is funded by social health insurance (UK, Taiwan, Philippines), private health insurance (USA, Japan), blended funding models (Australia, Canada, Singapore) and where substantial out-of-pocket expense remains (Peru). They were also at different phases of digital health maturity as per the Global Digital Health Monitor ( https://monitor.digitalhealthmonitor.org/map ) with countries being classified as overall in phase 3 (Japan, Peru), phase 4 (Philippines), and phase 5 (Australia, Canada, UK, USA, Singapore) with no data for Taiwan. Even so, none reported being fully compliant with the FAIR (Findable, Accessible, Interoperable and Reusable) Guiding Principles [ 14 ]. In the five countries which reported a risk management framework being used, two explicitly noted the use of the Five Safes framework [ 15 ] with others reporting general privacy rules and personal data protection laws being guiding principles.

Five countries reported on subnational collections of EHR data (Australia, Canada, Singapore, UK and USA) from a mix of city, state, and regional EHR data networks, with number of participants in the networks ranging from 35 thousand (Singapore) to close to 20 million (USA). The Australian electronic Practice based Research Network includes three EHR systems from 11 primary care sites and five hospitals [ 16 ]. The Canadian UTOPIAN data has >400,000 patients primary care EMR data patients from the Greater Toronto Area and beyond [ 17 ]. The Singapore National Healthcare Group Polyclinics EHR system has 35,051 patients. ( https://corp.nhg.com.sg/Pages/default.aspx ) The UK Oxford-Royal College of General Practitioners (RCGP) Research and Surveillance Centre (RSC) is one of the world's oldest sentinel networks covering 32% of the national population [ 18 ]. The USA DARTNet Institute (DI) collaboration includes 85 healthcare organizations, 13 academic medical centres, 3,000 clinicians, extensive IT professionals and electronic health record repositories that are working to build a national collection of electronic health record (EHR) data, claims data, and patient reported outcomes data [ 19 ].

The remaining four countries did not have established EHR data networks or could not easily access EHR data; they reported on specialised national data collections (Japan, Peru, Philippines and Taiwan), which usually had the Ministry or Department of Health as the data custodian. The Taiwan health information ecosystem was well-developed with the national health insurance data made available to clinicians via their the EMR systems on an almost real-time basis.

### 3.2. SDoH Taxonomies used in participating countries

Many countries are using or developing indicators consistent with the WHO SDoH taxonomy to better understand SDoH in their health ecosystem. However, the data models, data labels and metadata are not consistent across all countries. Table 1 summarises the SDoH taxonomy, metadata and data stewardship of SDoH data in the participating countries.

Many countries measure socio-economic status (SES) as an index of relevant SDoH indicators for a geographic or local government area, enabling the use of postcodes understand spatial distribution of SES. These indexes include the Socio-economic Indicators for Areas (Australia), Index of Multiple Deprivation (UK) or Local Index of Multiple Deprivation (Japan) [ 20 ]. Less well-resourced countries (Peru and Philippines) use a combination of income, employment and housing to assess SES.

Food security was included by four of nine countries: one country conceptualised it as part of social inequity, two included it as part of other national survey instruments/programmatic data collection, and one as part of routine data collection.

House ownership and early childhood data were well captured by all countries, noting that for many countries record early childhood data in separate specialised forms/databases such as the UK.

Discrimination was reported in various ways across the settings including as sense of belonging, having disability, physical, social, and workplace discrimination. Most of the countries reported having a universal health coverage system or a high proportion of the population with health insurance, as indicators of access and equity.

Social connectedness was reported as living arrangements ( *i.e.* , whether they live alone) in the subnational EHRs that record them (Australia, Canada, and Singapore) but not in any of the specialised national data collections. There was also inconsistency with some countries reporting social connectedness as referring to recording of “social benefit” or on “social conflict”. Social prescribing was reported mostly by the better resourced countries, where social prescribing is often covered by the health system (UK NHS, Singapore) or health insurance (Australia, Japan, Taiwan).

### 3.3. Quality of SDoH data from participating countries

Table 2 summarises the data completeness, conformance to a taxonomy or terminology, plausibility, and availability of prior quality assessment from the participating countries. Numbers are colour-coded based on a traffic-light convention (see table legend). There was variability in the level of availability of data even in countries which had more systematically explored and set standard for the indicators of interest.

#### 3.3.1. Demographic indicators

Both specialised national data collections and subnational EHR data were found to have a high proportion of completeness with regards to demographic details (age, gender) as well as rurality and regionality based on mainly postcode details, except for Singapore which is a city state. These variables for most countries were classified as ‘green’, meaning they are likely fit-for-purpose for use in practice.

#### 3.3.2. Social and equity-related indicators

The availability of social and equity-related data was variable. Education and occupation were poorly recorded (red) or not recorded, whereas income and residential care were well-recorded in only two (Japan and Taiwan) and three countries (Japan, Taiwan and UK) respectively. Socio-economic status was only available in three countries (Canada, Philippines and UK), all with a high degree of completeness (green).

#### 3.3.3. Clinical indicators

Among the five clinical-related indicators evaluated, smoking and obesity were the two where countries report some records being available. Even so, this was classified as red in five and six countries respectively of the nine countries. For the remaining three indicators, alcohol was classified as green in three countries (Philippines, Taiwan and UK) whereas mental health and substance use were poorly recorded in most of the countries. In the Philippines it was noted that substance use was not extracted for ethical reasons with concerns relating to legal implications raised by multiple other countries.

## 4. Discussion

The data collected and aggregated revealed patterns as well as discrepancies in EHR data availability, completeness, conformance, plausibility, and quality assessment for the SDoH data. The general conclusion is that all countries aspire to collect SDoH data, consistent with global developments within the WHO family. However, in practice the data are either not systematically or consistently collected, documented or easily accessible to primary or secondary users. The good news is that the data is being collected, albeit modestly, in both specialised national collections as well as in EHRs. This aligns with prior studies suggesting that SDoH data are often recorded in unstructured notes or not in electronic format raising challenges to extract and utilize these indicators in a meaningful way to inform clinical practice and population health management [ 21 ].

Demographic data such as age, gender and rurality are generally fit-for purpose. However, the availability and quality of data for education, occupation, income, socio-economic status, social and residential care varied considerably between countries. Clinical data such as those for smoking, obesity, alcohol use, mental health, and substance use were generally poorly collected, documented or accessible. There is a clear need for consistency and harmonisation across settings for the recording of these data to realise its potential in improving population health and clinical practice.

To enable general practice and family medicine to adequately address the SDoH, the health and welfare information ecosystem must enable the routine point-of-care collection of reliable, high-quality data to inform clinical decision-making and support this biopsychosocial (and therefore, multisectoral) model of care. To move towards this vision, it is essential that countries:

- Establish **standardised taxonomies, ontologies and reference standards** that enable the development of fit-for-purpose EHRs to support the collection of SDoH data that informs clinical decision-making;
- Define **indicators for SDoH** that are sufficiently specific to the local context (internally valid), yet comparable between subnational regions, as well as internationally (externally valid);
- Implement workforce **competencies, capability maturity and enablers** for the collection of SDoH data by citizens, health care professionals and organisations;
- Ensure that **SDoH data is routinely collected, assessed and managed** on a regular basis; and,
- Utilise SDoH data in clinical and public health interventions to improve health equity, build social capital, and improve citizens' digital inclusion.

### 4.1. Taxonomies: global standards and reference

Several taxonomies for SDoH data already exist and are in use. For example, the RCGP-RSC developed an ontological approach to curate SDOH indicators in EHRs, using SNOMED-CT, to guide the extraction of data from the sentinel network on a weekly basis before and around COVID-19 [ 22 , 23 ]. They found an increase in the recording of several SDoH indicators; namely issues related to homelessness, unemployment, mental health, harmful substance use and financial difficulties.

The USA's ‘Capturing Social and Behavioural Domains in EHRs’ study aims to identify domains and measures that capture the social determinants of health to inform the development of recommendations for meaningful use of EHRs. This report identifies specific domains to be considered by the Office of the National Coordinator, specifies criteria that should be used in deciding which domains should be included, identifies core social and behavioral domains to be included in all EHRs, and identifies any domains that should be included for specific populations or settings defined by age, socioeconomic status, race/ethnicity, disease, or other characteristics [ 8 ]. Studies such as these can be used to establish and institutionalise the collection and use of SDoH data at point of care. This will also guide the development of user-friendly and fit-for-purpose EHRs.

### 4.2. Indicators: global and local

While our analysis considered the data availability and quality for 16 indicators in nine countries, this is far from a comprehensive list of indicators that applies across countries. Countries that have yet to develop their own taxonomies and indicator sets can draw inspiration from those that have, while ensuring transferability and applicability in their own local context and between subnational regions – thus enabling comparisons to inform national policymaking efforts. At the international level, countries are already cooperating to harmonise data standards for cross-border data-sharing [ 24 , 25 ], recently resulting in the ‘International Patient Summary’ (IPS) which comprises a minimum shareable health dataset for each individual travelling across borders [ 26 ]. However, this should ideally include SDoH data to provide treating clinicians with a comprehensive picture of the individual's SDoH which has great influence on their care needs.

### 4.3. Implementing SDoH data collection: capability maturity and workforce competencies

Ensuring the uptake and utilisation of SDoH indicator data fields into health information systems requires the cooperation and coordination of health care professionals and organisations. National and regional standards for collecting SDoH data can be set by health ministries and defined as part of capability maturity in healthcare organisations, with enforcement by governing bodies and independent accrediting agencies [ 27 , 28 ]. With more secure and effective data linkage and better policies and regulatory frameworks to protect privacy and confidentiality, we can distribute the load of data collection across EHRs in routine care and periodic specialised data collections to enable the availability of SDoH data for the personalised care of individuals and populations. It is therefore also important to ensure that care professionals are familiar with how this information is to be recorded, highlighting the importance of workforce training in the implementation of SDoH data collection. Professional organisations can define professional competencies and offer training in these as part of continuous professional development programs. Several implementation toolkits exist for guiding this process [ 29 ]. On the other hand, EHRs would also need to be more versatile rather than be a barrier to recording of SDoH data as suggested in a previous qualitative study which found challenges in incorporating SDoH recording into existing EHR systems [ 30 ].

### 4.4. Real-world data quality collection, assessment and management

With digitalization and EHRs, we have the tools and infrastructure to collect and process data with greater precision. This increased granularity in “big digital data” requires greater automation in data quality assessment and management. Many frameworks are available to assess data quality [ 31 ].

An advantage of EHRs is the ability adopt a more nuanced approach through the collection of precise data. Using race/ethnicity as an example, individuals to specify their exact racial or ethnic identity ( *e.g.* , Laotian, Hmong, Vietnamese, Tamil, etc.). These can then be automatically categorised into broader categories ( *e.g.* , Asian, South Asian, etc), ultimately generalising to White, Black, Asian, Mixed, Other categories. This data aggregation can be applied to various SDoH parameters such as age, sex, education, race, and socio-economic variables, thus creating a data system that can adapt to specific needs and secondary use, while retaining the data that is already collected for accurate designation and understanding how racial identities intersect in the practice of precision medicine [ 32 ]. This can also support evaluation of the impact of new technologies on various targeted patient groups allowing for greater personalisation in practice [ 33 ].

### 4.5. Digital inclusion and citizen empowerment for people-centred care

The data generated from the inclusion of SDoH indicators in routine EHRs can go beyond informing clinical decision-making to provide researchers with valuable information with which to address the SDoH through integrated health and social care initiatives. Several frameworks and models outline how economic and social interventions can create both social and health impacts [ 34 , 35 ]. Addressing the SDoH also improves social capital in communities and neighbourhoods, which has been shown to impact health equity and outcomes [ 36 ], especially in younger populations [ 37 ].

However, to be effective, digital competencies are essential not only for care providers, but also care recipients and their carers. Digital inclusion is increasingly recognised as a social determinant of health [ 38 ], and several studies support the utility of digital tools and applications in meeting patient health information needs foster greater patient engagement, better support patients outside of clinics, and can improve health outcomes [ 39 ]. However, overreliance on digital tools could increase disparities between those with digital access and those without [ 40 ]. Building digital literacy skills, especially among the elderly will avoid creating a ‘digital divide’ [ 41 ].

## 5. Conclusion

The data confirms the WHO assessment in 2024 that since the establishment of the SDoH Commission in 2005, many countries still lack the data, information systems and capacity to adequately monitor progress on SDoH and related policies and practice to improve health and health equity.

A key challenge facing governments is the political will to develop and implement effective policies and interventions and the assessment of their impact on health equity. The key sociopolitical determinants are structural, such as inequitable economic systems, structural discrimination including intersecting racism and gender inequality, and weak societal infrastructure.

There is still much to be done to monitor the SDoH and their association with health and care, as well as monitoring of policies and interventions to address SDoH and health equity – the quintuple aims.
